# Identification and Characterization of *Salvia miltiorrhizain* miRNAs in Response to Replanting Disease

**DOI:** 10.1371/journal.pone.0159905

**Published:** 2016-08-02

**Authors:** Haihua Zhang, Weibo Jin, Xiaole Zhu, Lin Liu, Zhigui He, Shushen Yang, Zongsuo Liang, Xijun Yan, Yanfeng He, Yan Liu

**Affiliations:** 1 College of Life Sciences, Northwest A & F University, Yangling, 712100, China; 2 College of Life Sciences, Zhejiang Sci-Tech University, Hangzhou, 310018, China; 3 Tasly R&D Institute, Tasly Holding Group Co. Ltd, Tianjin, 300410, China; 4 Tianjin Tasly Modern TCM Resources Co., Ltd., Tianjin, 300402, China; Kunming University of Science and Technology, CHINA

## Abstract

Replanting disease is a major factor limiting the artificial cultivation of the traditional Chinese medicinal herb *Salvia miltiorrhiza*. At present, little information is available regarding the role of miRNAs in response to replanting disease. In this study, two small RNA libraries obtained from first-year (FPR) and second-year plant (SPR) roots were subjected to a high-throughput sequencing method. Bioinformatics analysis revealed that 110 known and 7 novel miRNAs were annotated in the roots of *S*. *miltiorrhiza*. Moreover, 39 known and 2 novel miRNAs were identified and validated for differential expression in FPR compared with SPR. Thirty-one of these miRNAs were further analyzed by qRT-PCR, which revealed that 5 miRNAs negatively regulated the expression levels of 7 target genes involved in root development or stress responses. This study not only provides novel insights into the miRNA content of *S*. *miltiorrhiza* in response to replanting disease but also demonstrates that 5 miRNAs may be involved in these responses. Interactions among the differentially expressed miRNAs with their targets may form an important component of the molecular basis of replanting disease in *S*. *miltiorrhiza*.

## Introduction

*Salvia miltiorrhiza* Bunge is a very popular traditional Chinese medicinal plant. The economic importance of *S*. *miltiorrhiza* results from the medicinal activity of extracts of its tuberous roots, which include tanshinones and salvianolic acid, and the herb has been used extensively to treat a variety of conditions, especially cardiovascular and cerebrovascular diseases [1–3]. Unfortunately, while several studies have described how to improve the major medicinal bioactive constituents of *S*. *miltiorrhiza* [4–8], little attention has been paid to *S*. *miltiorrhiza* yields and quality.

The cultivation area of *S*. *miltiorrhiza* in China has expanded over the years because of increased demands for the plant in the domestic and international markets. Environmental conditions, such as soil, climate, and altitude, play an important role in *S*. *miltiorrhiza* cultivation. Land suitable for planting *S*. *miltiorrhiza* is extremely limited. Consequently, replanting disease has become a major factor limiting the artificial cultivation of *S*. *miltiorrhiza* [9–11].

Replanting disease, also known as the continuous monoculturing problem, has been observed to induce sharps drop in the growth, development, yield, and quality of plants grown in a field where the same species had been grown over the past several years even under normal cultivation and management measures. This syndrome has been observed in approximately 70% of all medicinal plants with roots or rhizomes used as traditional medicine, including *Panax ginseng* [12], *Rhizomacoptidis* [13], and *P*. *notoginseng* [14]. Replanting disease presents serious influences on the sustainable development of medicinal plants and is a major issue that must be addressed in the industry.

Previous studies have suggested that changes in the soil properties, microbial population, and allelopathic autotoxicity promote replanting disease [15–16]. However, the molecular basis of the sensitivity of a species to its own exudate remains unknown. Yang et al. [13] recently studied the responses of genes of *Rehmannia glutinosa* to replanting disease during continuous cropping and obtained a global perspective of differentially transcribed genes and miRNAs in the plant.

Increasing lines of evidence suggest that miRNAs play an important role in plant stress, and that the expression profiles of most miRNAs involved in plant growth and development are significantly altered during environmental stress [17]. Given that replanting disease represents a form of stress, miRNAs may be involved in the replanting disease of *S*. *miltiorrhiza*.

We hypothesize that miRNA activity may underlie at least some of the problems associated with the continuous cropping of *S*. *miltiorrhiza*. To gain novel insights into the miRNA content of *S*. *miltiorrhiza* in replanting disease, we employed a high throughput parallel sequencing platform (Solexa sequencing) to generate two small libraries of the first-year (FPR) and second-year (SPR) plant roots of the herb. We applied this technology to achieve comparative profiles of miRNAs of the two libraries with the aim of identifying the miRNAs expressed differentially in FPR and SPR. We also identified and confirmed the responses of novel miRNAs to replanting disease using RT-PCR. The expression of miRNAs expressed differentially was confirmed using qRT-PCR, and the target genes were elucidated. In this study, a total of 110 known miRNAs were identified, and 7 novel miRNAs were discovered. Among these miRNAs,41 were differentially expressed between the two libraries. The targets of the differentially expressed miRNAs were also analyzed, and 7 targets from 5 miRNAs, including transcriptional regulation factors, proteases, and disease-resistance proteins, were eventually verified. The results suggest that the obtained miRNAs and their targets may play an important role in gene pathway responses to replanting disease.

## Materials and Methods

The experimental materials were planted at the GAP planting base of *S*. *miltiorrhiza* (Shangluo, Shanxi Province, China); this base belongs to Tasly R&D Institute, Tasly Holdings Group Co. Ltd., Tianjin, China. We obtained permission to conduct the study at this site and performed sample collection after authorization by Yan Liu, the official in charge of the GAP planting base. No endangered or protected species were involved in this study.

### Plants material and RNA extraction

The *S*. *miltiorrhiza* cultivar ‘violet flower *S*. *miltiorrhiza*’ was collected from the GAP planting base. The first-year crop was grown in a field where *S*. *miltiorrhiza* had not been planted for more than 5 years. A second group of plants was grown in a field where the same cultivar had been grown in the previous year. For convenience, we named members of the former group as first-year plants and members of the latter group as second-year plants. Root samples were obtained from five independent plants at the tuberous root expansion stage (Aug. 10, 2015), and their RNA content was extracted using an improved CTAB-LiCl method [18].

### Identification of novel miRNA in *S*. *miltiorrhiza*

Raw read sequences in the two libraries were combined into one small RNA library for novel miRNA prediction, and all reads matching the tRNA, rRNA, or conserved miRNA sequences with two mismatches were removed using Bowtie [19]. The remaining reads were mapped to the assembled transcript sequences (downloaded from http://bi.sky.zstu.edu.cn/Bio111/DsTRD/home.php) [20] using Bowtie with perfect matching [19]. With one end anchored 20 bp away from the mapped small RNA location, 120–360 bp sequences, each with an extension of 20 bp covering the small RNA region, were collected using a Perl script. Secondary structures of each sequence were predicted using RNAfold from the Vienna package (version 1.8.2) [21]. Under conditions similar to those proposed by Thakur *et al*. [22], a stem-loop structure with ≤ 3 gaps involving ≤ 8 bases at the small RNA location was considered a candidate miRNA precursor.

### Identification of replant-responsive miRNAs

The frequency of the miRNAs from two libraries was normalized to 1 million by total clean reads of miRNAs (RPM) in each sample. If the normalized read count of a given miRNA was zero, the expression value was modified to 0.01 for further analysis. The fold change between the SPR and FPR libraries was calculated as follows: fold change = log_2_(SPR/FPR). miRNAs with fold changes > 1 or < −1 and with *P*≤ 0.001 were respectively considered to be upregulated or downregulated in response to replanting disease. The *P*-value was calculated according to previously established methods [23].

### Prediction and validation of miRNA target genes

For identifying the miRNA targets, the FASTA files of *S*. *miltiorrhiza* transcriptome sequences were downloaded from the website http://bi.sky.zstu.edu.cn/DsTRD/downloads.php?codingRNAfa=codingRNA.fa. Following this, the online psRNAtarget program was first employed for target identification (http://plantgrn.noble.org/psRNA-Target/?function=3). Then degradome data of *S*. *miltiorrhiza* was downloaded from NCBI SRA database (accession number: SRR1557864) [24]. After removing the adaptor [24], clean reads were furtherly used for detecting the cleaved targets of miRNAs using CleaveLand pipeline [25].

### Quantitative RT-PCR analysis

Total RNA was treated with RNase-free DNase I (TaKaRa, Dalian, China) to remove genomic DNA. Forward primers for 5 selected miRNAs were designed based on the sequence of the miRNAs and are listed in S1 Table. The reverse transcription reaction was performed with the One Step PrimeScript miRNA cDNA Synthesis Kit (TaKaRa, Dalian, China) according to the manufacturer's protocol.

SYBR Green PCR was performed following the manufacturer’s instructions (Takara, Japan). In brief, 2 μl of cDNA template was added to 10 μl of 2× SYBR Green PCR master mix (Takara), 1 μM each primer, and ddH_2_O to a final volume of 20 μl. The reactions were amplified for 1min at 95°C, followed by 40 cycles of 95°C for 5 s and 60°C for 34 s. All reactions were performed in triplicate, and the controls (no template and no RT) were included for each gene. The threshold cycle (C_T_) values were automatically determined by the instrument. The fold-changes were calculated using 2 ^–Delta DeltaCt^ method, where DeltaDeltaCt = (Ct_*target*_ − Ct_*inner*_)_*treatment*_ − (Ct_*target*_ − Ct_*inner*_)_*control*_[26].

## Results

### Deep sequencing of sRNAs in *S*.*miltiorrhiza* root

**Fig 1:**
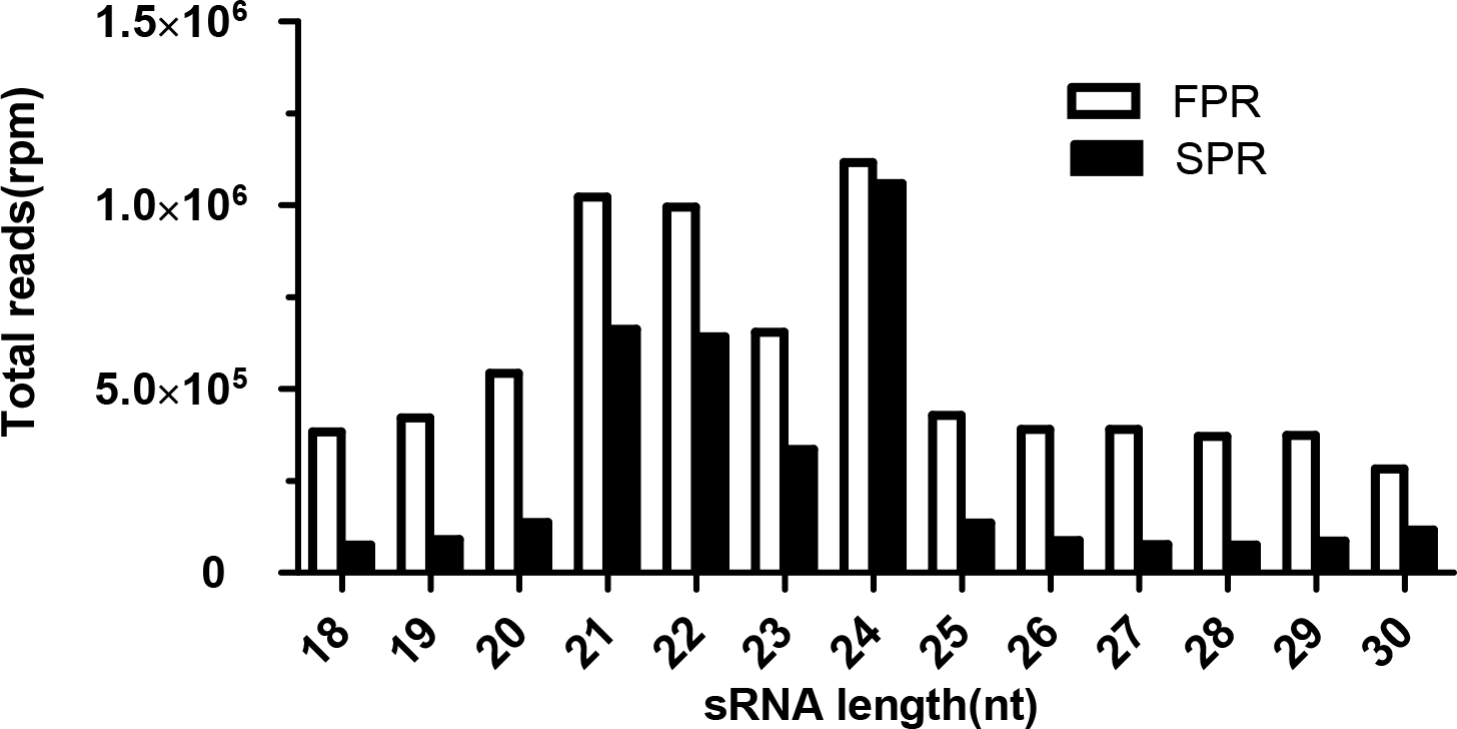
sRNA length distributions in *S*. *miltiorrhiza* FPR and SPR.

Two sRNA libraries were constructed from the root tissues of FPR and SPR to identify miRNAs responding to replanting disease in *S*. *miltiorrhiza* roots. The sequencing results were submitted to the GenBank SRA database (accession numbers: SRR3056582 and SRR3056449). A total of 14.73 million raw reads were generated from the two sRNA libraries. Low-quality tags and adaptor contaminations were removed to obtain 7,374,092 (representing 2,050,638 unique sequences) and 3,581,868 (representing 1,512,866 unique sequences) clean reads from the FPR and SPR libraries, respectively; these reads ranged from 18 nt to 30 nt in both groups. Sequences of about 21 and 24 nt in length were dominant in both libraries (Fig 1). This result is consistent with those presented in previous studies on other plant genera, such as *Arabidopsis* [27], *Oryza* [28], *Medicago* [29], and *Populus* [30].

### Identification of known miRNAs in *S*. *miltiorrhiza*

**Fig 2:**
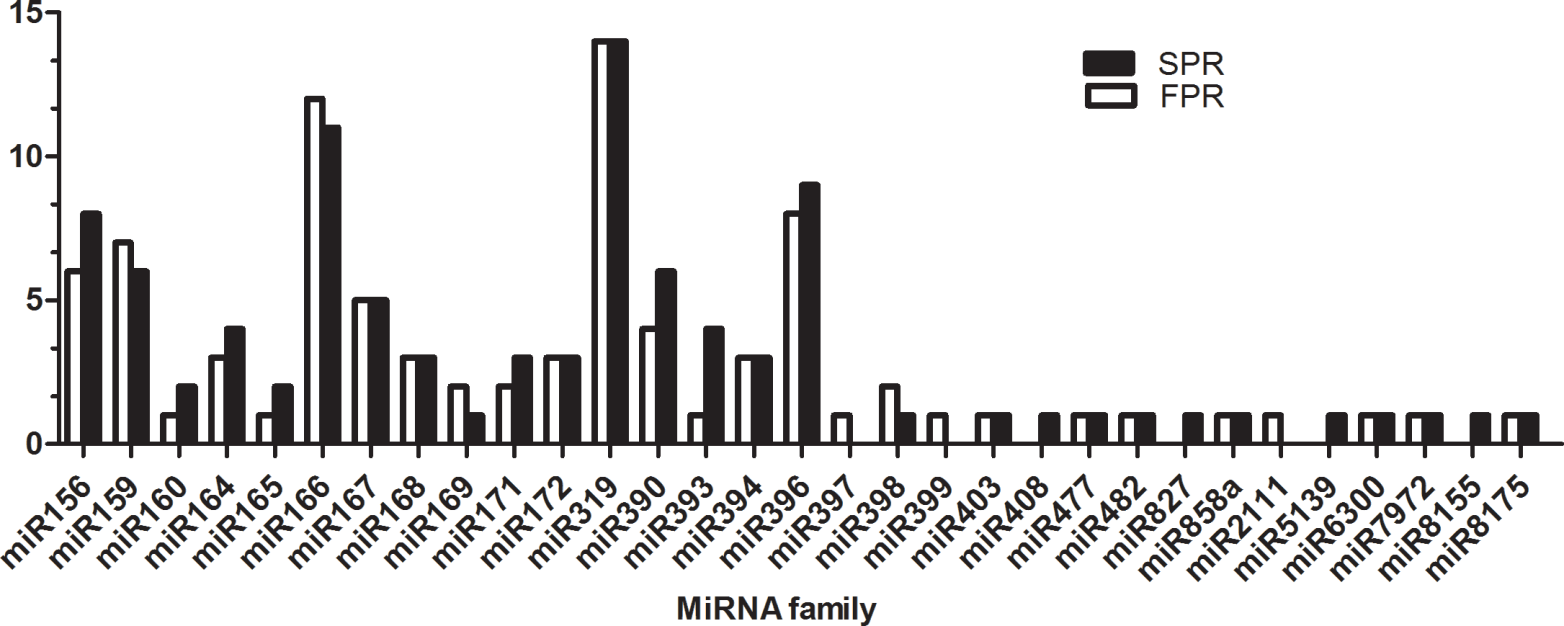
Number of known miRNA families in the FPR and SPR libraries.

Unique sRNA sequences were mapped to miRBase 21.0 with perfect matches using Perl script [31]. A total of 110 unique sequences belonging to 31 conserved miRNA families were identified in the FPR and SPR libraries (S2 Table). Among these sequences, 87miRNAs belonging to 27 families (except for miR408, miRNA827, miR5139 and miR8155) were detected in FPR, while 96 miRNAs belonging to 28 families (except for miR397, miR399 and miR2111) were detected in SPR. Between the two libraries, the more abundant sequences were found in the miR156, miR396, miR 319, and miR166 families. The miR319 family, which was composed of 14 members, dominated the sequences (Fig 2).

### Identification and validation of novel miRNAs in *S*. *miltiorrhiza*

sRNA reads that were homologous to known miRNAs and other non-coding sRNAs (Rfam 10) were excluded, and the secondary structures of the precursors of the remaining 19–24 nt sRNAs were analyzed using RNAfold software to determine novel miRNAs in *S*. *miltiorrhiza*. Precursors with canonical stem–loop structures were further analyzed using more stringent filters to ensure that they satisfied the criteria established by the research community [32, 33]. Thirty-two miRNAs candidates derived from 71 loci satisfied the screening criteria. The 32 putative miRNA precursors were then used to extract miRNA*s, which are considered strong evidence of DICER-LIKE-1 (DCL1)-derived products. Thirteen of the 32 miRNA candidates contained miRNA star (miRNA*) sequences identified from the same libraries; the remaining 19 candidates did not contain any miRNA* sequences (S3 Table). The 13 candidates with miRNA* sequences were considered to be novel *S*. *miltiorrhiza* miRNAs, whereas the19 remaining candidates without miRNA* sequences were considered potential *S*. *miltiorrhiza* miRNAs. The stem-loop structures and miRNA* sequences of the13 novel miRNAs are shown in S1 Fig.

**Fig 3:**
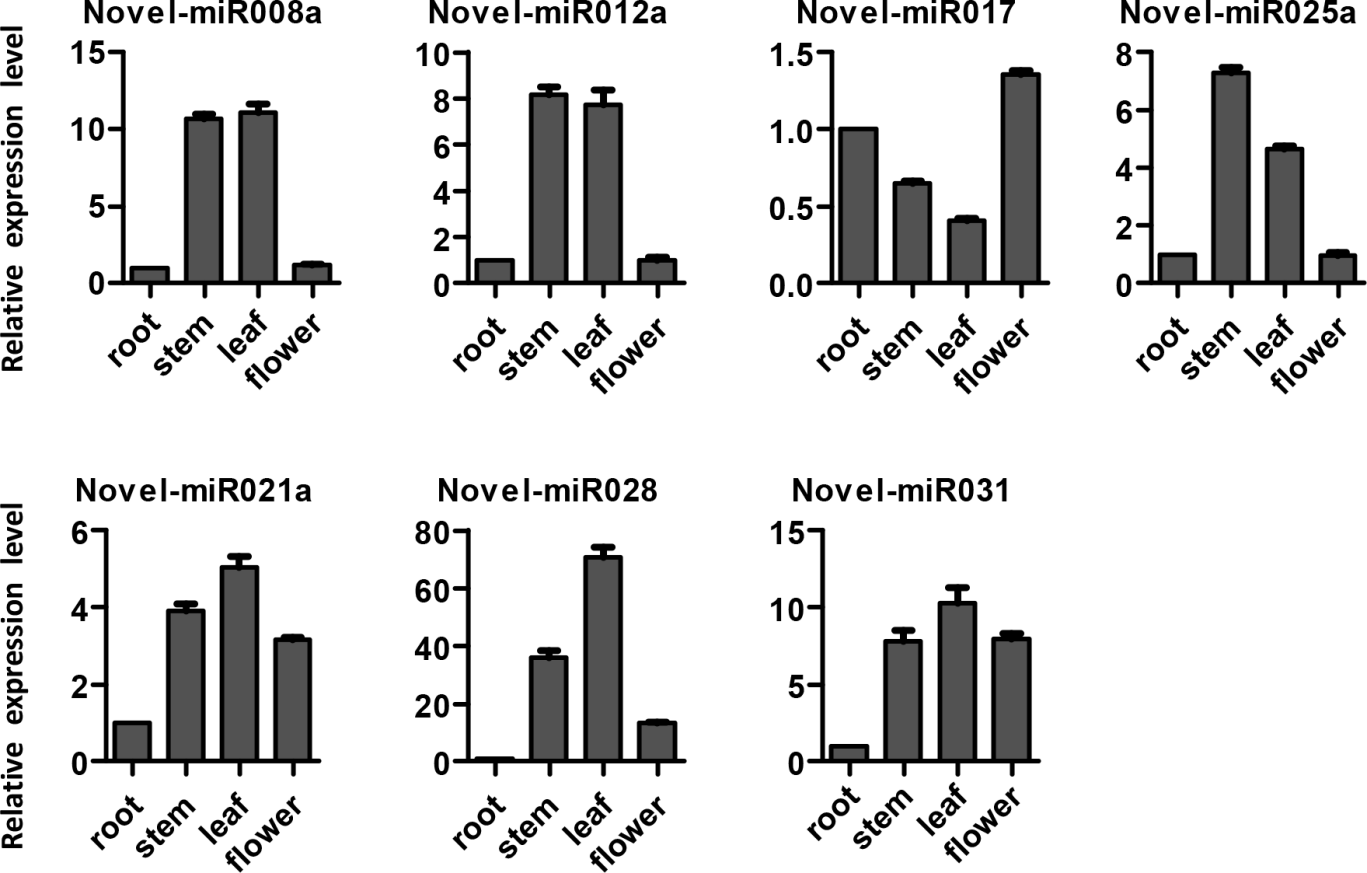
Expression levels of 7 novel miRNAs in the root, stem, leaf, and flower of *S*.*miltiorrhiza*.

RT-PCR was performed to validate the 13 novel miRNAs and determine their expression patterns in the root, stem, leaf, and flower of *S*. *miltiorrhiza*. The electrophoresis results indicated that the 7 novel miRNAs all showed amplification in the root, stem, leaf, and flower of *S*. *miltiorrhiza* (S2 Fig). Seven novel miRNAs were analyzed for tissue-specificity by qRT-PCR, and the results showed obvious tissue specificity. The expressions of all of the novel miRNA tissues, except that of miR017, were higher in the stem and leaf than in the root and flower. MiR021a, miR028, and miR031 expression levels were highest in the leaf, while miR025a expression levels were highest in the stem. The expression levels of miR031 in the root were lower by about 6 times than those in the stem, leaf, and flower of *S*. *miltiorrhiza*. miRNA017 gene expression was lower in the stem and leaf than in the root and flower; the highest expression of this gene was observed in the flower (Fig 3).The 7 novel miRNAs miR001a, miR008a, miR012a, miR017, miR021a, miR028 and miR031 were renamed as smi-miR35829, smi-miR35830, smi-miR35831, smi-miR35832, smi-miR35833, smi-miR35834 and smi-miR35835, respectively.

### Differential expression identification of known and novel miRNAs involved in response to replanting disease

To identify the miRNAs involved in the response of *S*. *miltiorrhiza* to replanting disease, differential expressions in the two libraries were estimated from the read counts determined through high-throughput sequencing. We selected RPMs that exceeded 1 and presented a P value of less than 0.001 from both libraries. miRNAs that met this condition and exhibited a log2 (SPR RPM/FPR RPM) fold-change higher than 1 were designated as upregulated, whereas those with a log2 (SPR RPM/FPR RPM) fold-change of less than −1 were designated as downregulated (S2 and S3 Tables).

The read counts of 110 known unique sequences from both libraries were retrieved to determine miRNAs responding to replanting disease. A total of 39 known miRNAs from 15 families and 2 novel miRNAs were differentially expressed in response to replanting disease (Table 1). Among these differentially expressed miRNAs, 38 were upregulated and only 3 were downregulated in the SPR library compared with those in the FPR library. Among these 41 sequences, the miR166, miR319 and miR396 families, which possessed 8, 5 and 5 members, respectively, were in a dominant position. Only pab-miR160a-like was absent from the FPR library.

**Table 1:** Differentially expressed miRNAs in the FPR and SPR libraries of *S*. *miltiorrhiza*.

miR_name	Mature Sequence(5'-3')	FPR_RPM	SPR_RPM	log2(SP/FP)	Pvalue
smi-miR156a-1	TGACAGAAGAGAGTGAGCAC	8.54	24.85	1.540938	6E-11
smi-miR156a-2	TGACAGAAGAGAGTGAGCAC	8.54	24.85	1.540938	6E-11
smi-miR156a-3	TGACAGAAGAGAGTGAGCAC	8.54	24.85	1.540938	6E-11
smi-miR159a	TTTGGATTGAAGGGAGCTCTA	691.88	2714.23	1.971949	0
aly-miR159b-3p-like	TTTGGATTGAAGGGAGCTCTT	0.81	58.91	6.184447	3.05E-93
pab-miR160a-like	TGCCTGGCTCCCTGTATGCCA	0.01	2.79	8.124121	9.12E-06
smi-miR164a-1	TGGAGAAGCAGGGCACGTGCA	12.61	112.51	3.157413	8.1E-110
smi-miR164a-2	TGGAGAAGCAGGGCACGTGCA	12.61	112.51	3.157413	8.1E-110
ath-miR165a-3p-like	TCGGACCAGGCTTCATCCCCC	2.31	6.7	1.536268	0.000636
smi-miR166a-3p-1	TCGGACCAGGCTTCATTCCCC	20432.21	47641.34	1.221369	0
smi-miR166a-3p-2	TCGGACCAGGCTTCATTCCCC	20432.21	47641.34	1.221369	0
smi-miR166a-3p-3	TCGGACCAGGCTTCATTCCCC	20432.21	47641.34	1.221369	0
smi-miR166a-3p-4	TCGGACCAGGCTTCATTCCCC	20432.21	47641.34	1.221369	0
smi-miR166a-5p-4	GGAATGTTGTTTGGCTCGAGG	10.85	4.19	-1.37267	0.000252
osa-miR166e-3p-like	TCGAACCAGGCTTCATTCCCC	24.55	115.86	2.238588	8.35E-76
smi-miR166h-3p	TCTCGGACCAGGCTTCATTCC	495.25	1758.58	1.828182	0
smi-miR166a-5p	GGAATGTTGTCTGGCTCGAGG	99.81	33.5	-1.57502	6.67E-36
smi-miR167b-5p	TGAAGCTGCCAGCATGATCTG	3.93	34.62	3.139005	8.75E-35
smi-miR168a-5p-1	TCGCTTGGTGCAGGTCGGGAA	39.06	200.73	2.361492	4.3E-139
smi-miR168a-5p-2	TCGCTTGGTGCAGGTCGGGAA	39.06	200.73	2.361492	4.3E-139
smi-miR171a-3p	TGAGCCGAACCAATATCACTC	200.57	456.19	1.185529	1.8E-114
smi-miR319a-3p-1	TTGGACTGAAGGGAGCTCCC	166.39	424.36	1.35072	4.1E-131
smi-miR319a-3p-2	TTGGACTGAAGGGAGCTCCC	166.39	424.36	1.35072	4.1E-131
smi-miR319a-3p-3	TTGGACTGAAGGGAGCTCCC	166.39	424.36	1.35072	4.1E-131
smi-miR319a-3p-4	TTGGACTGAAGGGAGCTCCC	166.39	424.36	1.35072	4.1E-131
smi-miR319a-3p-5	TTGGACTGAAGGGAGCTCCC	166.39	424.36	1.35072	4.1E-131
smi-miR390a-5p-1	AAGCTCAGGAGGGATAGCGCC	3.25	8.1	1.317482	0.000892
smi-miR390a-5p-2	AAGCTCAGGAGGGATAGCGCC	3.25	8.1	1.317482	0.000892
smi-miR390a-5p-3	AAGCTCAGGAGGGATAGCGCC	3.25	8.1	1.317482	0.000892
smi-miR394a-5p-1	TTGGCATTCTGTCCACCTCC	7.87	16.75	1.089726	4.31E-05
smi-miR394a-5p-2	TTGGCATTCTGTCCACCTCC	7.87	16.75	1.089726	4.31E-05
smi-miR394a-5p-3	TTGGCATTCTGTCCACCTCC	7.87	16.75	1.089726	4.31E-05
smi-miR396b-5p-1	TTCCACAGCTTTCTTGAACTT	62.79	317.15	2.336559	1.3E-215
smi-miR396b-5p-2	TTCCACAGCTTTCTTGAACTT	62.79	317.15	2.336559	1.3E-215
smi-miR396a-5p	TTCCACAGCTTTCTTGAACTG	1408.85	6879.65	2.287817	0
smi-miR396c-5p	TTCCACAGCTTTCTTGAACTA	143.07	1340.36	3.227827	0
gma-miR396e-like	TTCCACAGCTTTCTTGAACTGT	0.54	3.35	2.63313	0.000549
har-miR403a-like	TTAGATTCACGCACAAACTCG	58.99	227.26	1.945801	1.2E-121
smi-miR482a	TTTCCAACTCCACCCATTCCTA	24.55	222.79	3.18189	2.7E-217
smi-miR35832	GGTGCAATGGGCGAACGCCGAGG	50.85	15.36	-1.72707	2.13E-21
smi-miR35835	TTTCCAATGCCGCCCATACCGA	327.09	1715.59	2.390945	0

**Fig 4:**
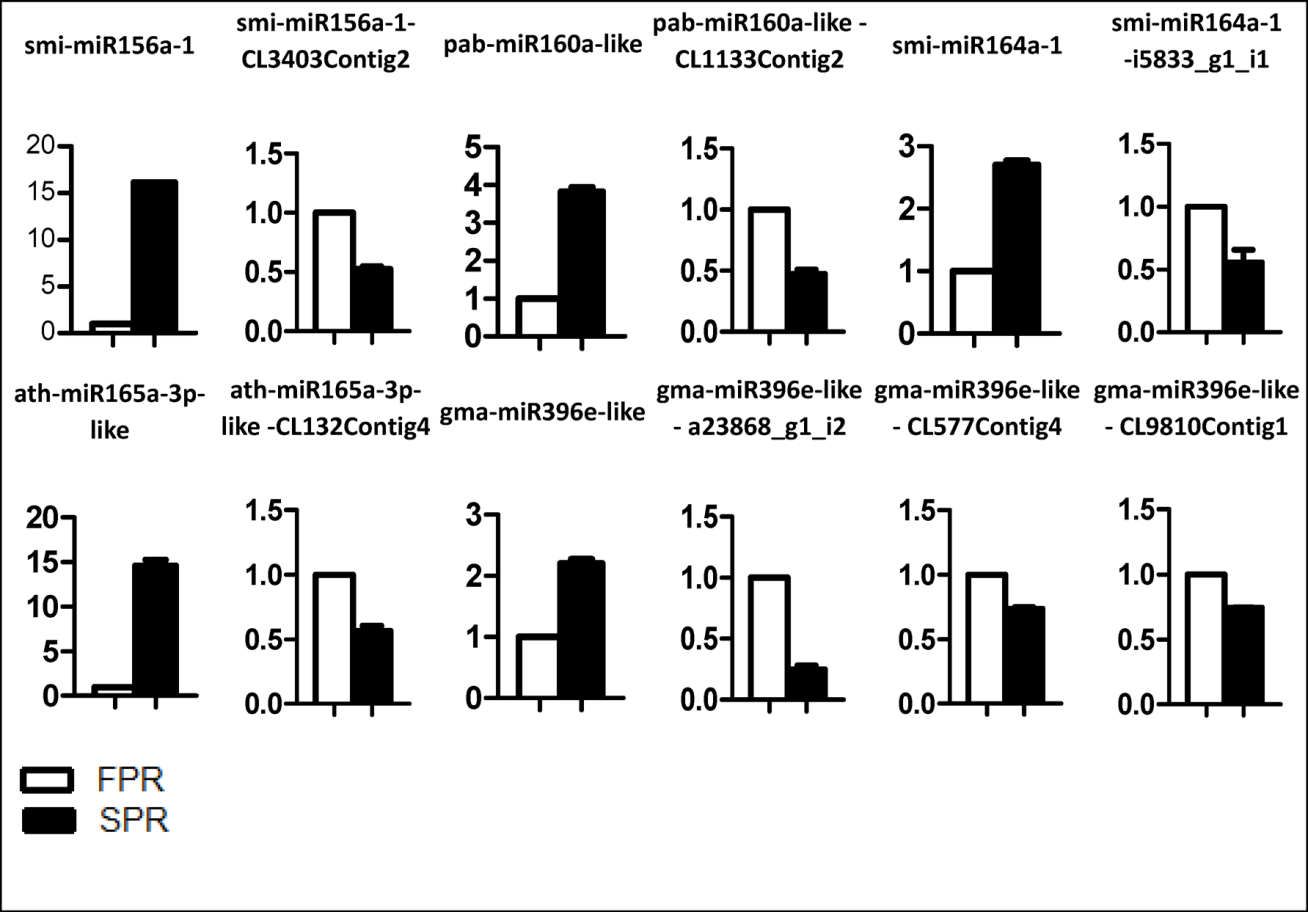
Verification of differential expression miRNAs and their targets by qRT-PCR in FPR and SPR.

The expression levels of the differentially expressed miRNAs were reanalyzed using qRT-PCR. The 5 miRNAs (smi-miR319a-3p-1, smi-miR319a-3p-2, smi-miR319a-3p-3, smi-miR319a-3p-4 and smi-miR319a-3p-5) could not be amplified. The abundances estimated by sequencing and qRT-PCR analysis were consistent for31 of the 36 miRNAs (the exceptions were smi-miR166a-5p-4, smi-miR394a-5p-1, smi-miR394a-5p-2, smi-miR394a-5p-3 and smi-miR35832 (Fig 4 and S3 Fig). However, the fold-changes obtained from the qRT-PCR data were much higher than those estimated from the high-throughput sequencing data, presumably because of differences in the sensitivity and specificity of the experimental approaches.

### Analysis and validation of target genes

The psRNAtarget program was used to predict the targets of 31 replant-responsive miRNAs, only 41 CDSs were targeted by 12 miRNA families (S4 Table). CleaveLand pipeline was performed for the second screening of the targets (S4 Fig). The results showed that 20 CDSs were targeted by 10 miRNA families except for miR403 (Table 2). The expression profiles of these 20 CDSs were furtherly determined using qRT-PCR. The result showed that PCR failed to amplify three targets containing g7803_g1_i2, CL765Contig5 and CL2Contig108. Among remaining 18 target cDNAs, SPL13 (CL3403Contig2), ARF18-like (CL1133Contig2), NAC100-like (i5833_g1_i1), athb-15-like (CL132Contig4) and three members of the growth-regulating factor family (a23868_g1_i2, CL577Contig4 and CL9810Contig1), which were targeted by smi-miR156a-1, pab-miR160a-like, smi-miR164a-1, ath-miR165a-3p-like and gma-miR396e-like, respectively, were significantly downregulated in SPR, and exhibited a negative relationship to the expression of the 5 miRNAs (Fig 4). However, GRF4-like (CL1008Contig3), disease resistance protein rpm1-like (CL3Contig201) and hypothetical protein MIMGU_mgv1a023991mg(CL2Contig108), which were targeted by gma-miR396e-like, smi-miR482a and novel miRNA smi-miR35835, respectively, were significantly upregulated, and exhibited a consistent expression pattern with that of these miRNAs (S3B Fig). In addition, no significant differential expression in SPR was observed in the remaining 8target cDNAs (S3A Fig). Therefore, the results strongly suggested that the smi-miR156a-1, pab-miR160a-like, smi-miR164a-1, ath-miR165a-3p-like and gma-miR396e-like may be involved in the responses to replanting disease in *S*. *miltiorrhiza*.

**Table 2:** Identified targets of differential expression miRNAs in *S*. *miltiorrhiza* by degradome sequencing.

miR_name	Target_Acc.	Length	Target_Desc.
smi-miR156a-1	CL1799Contig3	2055	squamosa promoter-binding-like 12-like, SPL12L
smi-miR156a-1	CL3403Contig2	1281	squamosa promoter-binding-like 13, SPL13
smi-miR159a	CL542Contig9	1294	transcription factor gamyb-like isoform x1
pab-miR160a-like	g7803_g1_i2	2690	auxin response factor 18-like, ARF18-like
pab-miR160a-like	CL1133Contig2	2925	auxin response factor 18-like, ARF18-like
pab-miR160a-like	CL16970Contig1	1429	auxin response factor 18-like, ARF18-like
smi-miR164a-1	i5833_g1_i1	906	NAC domain-containing protein 100-like, NAC100
ath-miR165a-3p-like	CL132Contig4	2174	homeobox-leucine zipper protein athb-15-like
smi-miR167b-5p	a28228_g1_i3	933	mitochondrial substrate carrier family protein b-like
smi-miR167b-5p	k12130_g1_i1	1378	mitochondrial substrate carrier family protein b-like
smi-miR167b-5p	CL1585Contig3	1545	mitochondrial substrate carrier family protein b-like
smi-miR168a-5p-1	CL600Contig1	3706	argonaute 1
gma-miR396e-like	a23868_g1_i2	772	growth-regulating factor 3-like, GRF3-like
gma-miR396e-like	e14536_g1_i4	1078	growth-regulating factor 4-like, GRF4-like
gma-miR396e-like	CL1008Contig3	1155	growth-regulating factor 4-like, GRF4-like
gma-miR396e-like	CL577Contig4	2862	growth-regulating factor 4-like, GRF4-like
gma-miR396e-like	CL1003Contig3	3662	6-phosphofructokinase 3-like
gma-miR396e-like	CL9810Contig1	1625	growth-regulating factor 2-like, GRF2-like
smi-miR482a	CL3Contig201	422	disease resistance protein rpm1-like
smi-miR35835	CL2Contig108	1770	hypothetical protein MIMGU_mgv1a023991mg

## Discussion

### Identification of *S*. *miltiorrhiza* miRNAs by high-throughput sequencing

Identification of miRNAs in medicinal plants, such as *R*. *glutinosa* [13], *Panax ginseng* [34], and *Pinellia pedatisecta* [35], using high-throughput sequencing or miRNA arrays has previously been reported. High-throughput sequencing is particularly useful for identifying involved abiotic and biotic stress responses [36, 37]. In this study, miRNA libraries constructed from the FPR and SPR were used to identify novel and replant-responsive miRNAs in *S*. *miltiorrhiza*, and their mechanisms of action were subsequently investigated. Among the miRNAs identified using high-throughput sequencing, 65% of those already known were expressed at low levels (less than 10 raw reads; S1 Table). This finding suggests that high-throughput sequencing is a powerful tool for identifying poorly expressed miRNAs in plants. The miR160, miR165/166, and miR396 families, which are believed to target auxin response factor (ARF), homeodomain leucine zipper (HD-ZIP III), and growth-regulating factor (GRF) transcription factors, respectively, were abundantly represented in both libraries. By comparing the expression levels of all members of a miRNA family, dominant members, such as miR166 and miR396, may be found. These dominant members may perform key regulatory roles in response to stress. Some family members, such as Smi-miR166a/b/c/e/f/g/h/I in the miR166 family, exhibited comparable expression levels, thereby indicating that several members of a family may exert synergistic effects in the relevant regulatory network.

With the discovery of miRNAs as ubiquitous regulators of plant growth and development, including nearly all biological, metabolic, and stress processes, researchers have focused on miRNAs in *S*. *miltiorrhiza*. However, given that the complete genome of *S*. *miltiorrhiza* is currently unavailable, the discovery of miRNAs from *S*. *miltiorrhiza* is relatively limited.

Xu *et al*. [24] studied tissue-specific miRNAs i*n S*.*miltiorrhiza* and identified 164 miRNAs after redundancy elimination; of these miRNAs, 28 were known, 22 were novel, and another 114 miRNAs were considered homologous with known miRNAs. Some of these 114 miRNAs were 1–4 bases to the left or right of known miRNA sequences or differed from known miRNA sequences in terms of one base. In this study, we identified 117 miRNAs, 110 of which belonged to 31 known families and 7 of which were novel miRNAs. While twenty-three known miRNAs were consistently identified between the present study and Xu *et al*.’s research [24] (S5 Table), no novel miRNA were identified between both works, likely because assembled transcriptome sequences were used as reference RNAs for the miRNA precursors analysis in our study whereas ESTs were used in Xu *et al*.’s work.

### Medicinal plant miRNAs responding to replanting

Yang et al. [13] identified 16 miRNAs from 13 miRNA families, including 2 novel miRNAs, responding to *R*. *glutinosa* replanting disease. In this study, we identified 31 miRNAs from 14 miRNA families, including 1 novel miRNA responding to *S*. *miltiorrhiza* replanting disease. In addition, three replant-responsive miRNAs, namely sim-miR156a and sim- miR160a, were identified both in *S*. *miltiorrhiza* and *R*. *glutinosa* (S6 Table), suggesting that these 2 miRNAs might have important role in respons to replanting disease.

### Potential targets of replanting disease-associated miRNAs

Several studies have shown that miR165 [38], miR396 [39], miR164 [40], miR160 [41], and miR156 [42] are related to the growth and development of plant root. MiR160 is a major regulator of root growth and gravitropism, negatively regulating the target auxin response factors 10 (ARF10), ARF16, and ARF17 [42]. Transgenic plants overexpressing the miR160c gene, which downregulates the expression levels of ARF10 and ARF16 mRNAs, show short roots lacking gravity-sensing capability as well as enlarged tumor-like apices [43]. Overexpression ARF17, another target of miR160, has been observed to lead to the reduction of primary root length in *A*. *thaliana* [44]. MiR164 negatively regulates lateral root development by targeting NAC1 [45, 46]. Transgenic *Arabidopsis* overexpressing *ZmNAC1* leads to increased numbers of lateral roots in comparison with wild-type plants, and miR164 negatively regulates *ZmNAC1* [47]. Overexpression of miR166/165 downregulates target HD-ZIP IIIs and promotes root growth [38]. MiR396 targets seven growth-regulating factor (GRF) genes to regulate root development [48]. Thus, miR396a knock-down lines develop longer roots than wild-type plants, whereas miR396a and miR396b over-expressing lines and plants overexpressing miR396-resistant *AtGRF1* or *AtGRF3* exhibit shorter roots [39,48]. MiR156 regulates lateral roots by targeting SPL3, SPL9, and SPL10 in *Arabidopsis* [42].

**Fig 5:**
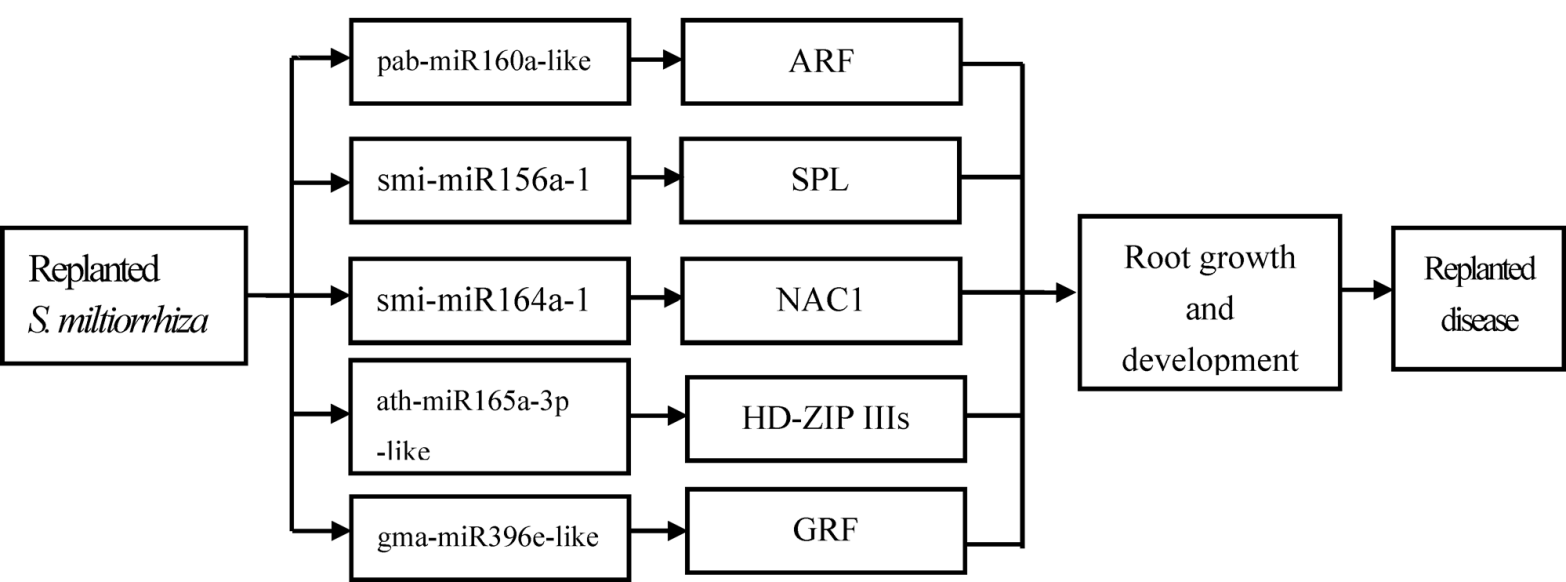
Proposed regulatory mechanism of miRNAs in replanted *S*. *miltiorrhiza*.

In our studies, smi-miR156a-1, pab-miR160a-like, smi-miR164a-1, ath-miR165a-3p-like, and gma-miR396e-like were upregulated in SPR and negatively regulated the targets of SPL13, ARF18-like, NAC100-like, homeobox-leucine zipper protein athb-14-like, and GRF3-like, respectively. Combining with the phenotype of poor growth in second-year crop, our results revealed that replanting disease upregulates the expressions of smi-miR156a-1, pab-miR160a-like, sim-miR164a-1, ath-miR165a-3p-like, and gma-miR396e-like. Correspondingly, their target genes including *SPL13*, *ARF18-like*, *NAC100-like*, *athb-14-like and GRF3-like* which were related to root growth were downregulated and then might be presented a growth inhibition (Fig 5).

## Conclusion

Our study provides a global perspective of differential miRNA expression in *S*. *miltiorrhiza* in response to replanting disease for the first time. We identified 110 known and 7 novel miRNAs, of which 5 known miRNAs were expressed differentially in the first- and second-year crops. Further investigation revealed that the target genes of differentially expressed miRNAs were related to root growth and development. Combining with the phenotype of poor growth in second-year crop, our results revealed that replanting disease upregulates the expressions of smi-miR156a-1, pab-miR160a-like, smi-miR164a-1, ath-miR165a-3p-like, and gma-miR396e-like. Correspondingly, their target genes including *SPL13*, *ARF18-like*, *NAC100-like*, *athb-14-like and GRF3-like* which were related to root growth were downregulated and then might be presented a growth inhibition.

## Supporting Information

S1 Fig Secondary structures of the novel miRNAs. (PDF)

S2 Fig RT-PCR products of the 7 novel miRNAs in the root, stem, leaf, and flower of *S*. *miltiorrhiza*. (PDF)

S3 Fig Comparison of the expression levels from differential expression miRNAs and the targets between FPR and SPR using qRT-PCR. A) The miRNAs were significantly upregulated and the targets were no significant differential expression in SPR; B) The miRNAs and the targets were both significantly upregulated in SPR;C) Differential expression miRNAs without matching targets.
(PDF)

S4 Fig Target plots (t-plots) of miRNAs and their targets. The red dot indicate the cleavage sites.
(PPTX)

S1 Table Primers used in this study. (XLSX)

S2 Table Identification of known miRNAs in *S*. *miltiorrhiza*. (XLSX)

S3 Table Identification of novel miRNAs in *S*. *miltiorrhiza*. (XLSX)

S4 Table Target prediction for replant-responsive miRNA *via psRNAtarget* pipeline (XLSX)

S5 Table Comparison of *S*. *miltiorrhiza* miRNAs between Xu et al. [38] and the present study. (XLSX)

S6 Table Comparison of miRNAs responding to replanting disease between *S*. *miltiorrhiza* and *R*. *glutinosa*. (XLSX)
