# Supplementary material for: Identification and Characterization of Salvia miltiorrhizain miRNAs in Response to Replanting Disease
Source: PLoS One. 2016 Aug 2;11(8):e0159905. doi: 10.1371/journal.pone.0159905 (PMC4970794; doi:10.1371/journal.pone.0159905)
Supplement: S1 Fig — (PDF) [file pone.0159905.s001.pdf]

```
Secondary structures of 7 novel miRNAs

1.Novel-miR008a
pri_seq                                      aguugcagagagggaugauuauggaaauguuaugaguugugugaugggcgaucucgucucuucugaguuagguucuucuaacacuauaacauuuccguaaucaucucucucagcagcuugug
t00706007_x1                                 ........agagggaugauuauggaaaug.............................................................................................  0
t00166860_x1                                 .........gagggaugauuauggaaaugu............................................................................................  0
t00691285_x1                                 ..........agggaugauuauggaaaug.............................................................................................  0
t01571169_x3                                 ..........agggaugauuauggaaaugu............................................................................................  0
t04054968_x89                                ..........agggaugauuauggaaauguu...........................................................................................  0
t01832491_x1                                 ..........agggaugauuauggaaauguuau.........................................................................................  0
t00492783_x9                                 ..........agggaugauuauggaaauguuaug........................................................................................  0
t03505451_x6                                 ......................................................................................uaacauuuccguaaucaucucucu............  0
t01431687_x1                                 ........................................................................................acauuuccguaaucaucucucu............  0
t03878032_x3                                 .........................................................................................cauuuccguaaucaucuc...............  0
t02643744_x15                                .........................................................................................cauuuccguaaucaucucu..............  0
t01415527_x42                                .........................................................................................cauuuccguaaucaucucuc.............  0
t02322145_x1466                              .........................................................................................cauuuccguaaucaucucucu............  0
t01706810_x6                                 ..........................................................................................auuuccguaaucaucucucu............  0
t03679060_x1                                 ..........................................................................................auuuccguaaucaucucucuc...........  0
t01928349_x2                                 ...........................................................................................uuuccguaaucaucucucuca..........  0

----      a                          g   u  gu  u    gau-    cc
    aguugc gagAGGGAUGAUUAUGGAAAUGUUau agu gu  ga gggc    cucg  u
    |||||| |||||||||||||||||||||||||| ||| ||  || ||||    ||||  c
    ucgacg cUCUCUCUACUAAUGCCUUUACaaua uca ca  cu cuug    gagu  c
gugu      a                          -   -  au  u    auug    cu




2.Novel-miR012a
pri_seq                                      uuuucucacucacauccucaaacccuuccaugcuccuuccggagagcgcugugugcucuaauaacagccuacauucgucuucugaaaggagcccuag
t04424978_x5                                 .....................................uccggagagcgcugugugcuc.......................................   0
t00447744_x2                                 ...................................................................ccuacauucgucuucugaaagg........   0
t00792533_x15                                ....................................................................cuacauucgucuucugaaagg........   0
t00585376_x1                                 ....................................................................cuacauucgucuucugaaaggagc.....   0
t03843385_x9                                 .....................................................................uacauucgucuucugaaagga.......   0
t02093624_x2                                 ......................................................................acauucgucuucugaaagga.......   0
t04043063_x1                                 .......................................................................cauucgucuucugaaaggagc.....   0

uuuucucacucacauccucaaacccuuuucu       C     A   C-    U   cua
                               gcuccUU CGGAG GCG  UGUG GCu   a
                               ||||||| ||||| |||  |||| |||
                               cgaGGAA GUCUU UGC  ACAU cga   a
--------------------------gaucc       A     C   UU    C   caa



3.Novel-miR017
pri_seq                                      uuuguggugcaaugggcgaacgccgagguggugucaguccaaccccggcguugucauugcaccacucacaaucauauguugaauu
t01569569_x12                                .....ggugcaaugggcgaacgcc.............................................................      0
t01439413_x2                                 .....ggugcaaugggcgaacgccga...........................................................      0
t00262521_x5                                 .....ggugcaaugggcgaacgccgag..........................................................      0
t02681595_x472                               .....ggugcaaugggcgaacgccgagg.........................................................      0
t00294883_x23                                .....ggugcaaugggcgaacgccgaggu........................................................      0
t04117279_x1                                 ......gugcaaugggcgaacgccgagg.........................................................      0
t04216510_x3                                 .......ugcaaugggcgaacgccgaggu........................................................      0
t00415283_x1                                 .........................................accccggcguugucauugcacc......................      0
t04392981_x8                                 ...........................................cccggcguugucauugcacc......................      0
t04020743_x6                                 ...........................................cccggcguugucauugcaccac....................      0
t01161351_x3                                 ............................................ccggcguugucauugcac.......................      0
t02013798_x99                                ............................................ccggcguugucauugcacc......................      0
t02593924_x49                                ............................................ccggcguugucauugcacca.....................      0
t00494785_x433                               ............................................ccggcguugucauugcaccac....................      0
t03469120_x8                                 ............................................ccggcguugucauugcaccacu...................      0
t04058079_x2                                 .............................................cggcguugucauugcacc......................      0
t03050152_x3                                 .............................................cggcguugucauugcacca.....................      0
t04099199_x2                                 .............................................cggcguugucauugcaccac....................      0

-----------------uuu            G G       A   -  ug
                    guGGUGCAAUGG C AACGCCG GGu gg  u
                    |||||||||||| | ||||||| ||| ||  c
                    CACCACGUUACU G UUGCGGC cca cc  u
uuaaguuguauacuaacacu            - -       C   a  ug



4.Novel-miR021a
pri_seq                                      cuacaucgccggcgaucaccagaaucaaagugucgcccaucacgcaagauucuggugauuccgaugaugugguu
t02380915_x3                                 .....ucgccggcgaucaccaga...................................................  0
t00621995_x4                                 .....ucgccggcgaucaccagaau.................................................  0
t03696389_x20                                .....ucgccggcgaucaccagaauca...............................................  0
t00117061_x2                                 ................................................auucuggugauuccgaugaug.....  0

--        C   C             Aaa-   ucg
  cuacaUCG CGG GAUCACCAGAAUC    gug   c
  |||||||| ||| |||||||||||||    |||
  gguGUAGU GCC UUAGUGGUCUUAg    cac   a
uu        A   -             acga   uac
```

```
5.Novel-miR025a
pri_seq                                      augugugauuugcucgggcuuucagaauguaauaaaaacaaagcuaugaauguguuuauuguuacuguaugguggcgaugaugacacucuuauuugucgacgacauuguuacauucugagagcuugagcuaaucacucauuc
t04404737_x3                                 ..........ugcucgggcuuucagaaug.................................................................................................................      0
t00590005_x13                                ..........ugcucgggcuuucagaaugu................................................................................................................      0
t03327390_x8                                 ..........ugcucgggcuuucagaaugua...............................................................................................................      0
t00088296_x4                                 ..........ugcucgggcuuucagaauguaaua............................................................................................................      0
t00305519_x1                                 .............ucgggcuuucagaaugua...............................................................................................................      0
t01368102_x9                                 .............ucgggcuuucagaauguaa..............................................................................................................      0
t00915922_x62                                .............ucgggcuuucagaauguaau.............................................................................................................      0
t01524831_x118                               .............ucgggcuuucagaauguaaua............................................................................................................      0
t00852332_x1                                 .............ucgggcuuucagaauguaauaa...........................................................................................................      0
t02545874_x1                                 .............ucgggcuuucagaauguaauaaaa.........................................................................................................      0
t03560418_x9                                 ..............cgggcuuucagaauguaau.............................................................................................................      0
t02150056_x17                                ..............cgggcuuucagaauguaaua............................................................................................................      0
t02451968_x20                                ..............cgggcuuucagaauguaauaa...........................................................................................................      0
t03365243_x12                                ..............cgggcuuucagaauguaauaaa..........................................................................................................      0
t00196404_x3                                 ...............gggcuuucagaauguaaua............................................................................................................      0
t03838623_x7                                 .........................................................................................................uuguuacauucugagagcuugagc.............      0
t03476293_x11                                ...........................................................................................................guuacauucugagagcuugag..............      0
t02931768_x5                                 ............................................................................................................uuacauucugagagcuuga...............      0
t02637741_x4                                 ............................................................................................................uuacauucugagagcuugag..............      0
t03122587_x2904                              ............................................................................................................uuacauucugagagcuugagc.............      0
t01233064_x70                                ............................................................................................................uuacauucugagagcuugagcu............      0
t00649218_x126                               .............................................................................................................uacauucugagagcuugagcu............      0
t02383521_x11                                ..............................................................................................................acauucugagagcuugagc.............      0
t03460511_x140                               ..............................................................................................................acauucugagagcuugagcu............      0
t02881657_x165                               ..............................................................................................................acauucugagagcuugagcua...........      0
t01968315_x36                                ...............................................................................................................cauucugagagcuugagcu............      0
t04411746_x47                                ...............................................................................................................cauucugagagcuugagcua...........      0
t01154153_x897                               ...............................................................................................................cauucugagagcuugagcuaa..........      0

--   u      u                        aaacaaagcu--    au     --          g
  aug gugauu gcUCGGGCUUUCAGAAUGUAAUAa            auga  guguu  uauuguuacu u
  ||| |||||| ||||||||||||||||||||||||            ||||  |||||  ||||||||||
  uac cacuaa CGAGUUCGAGAGUCUUACAUUguu            uauu  cacag  guagcggugg g
cu   u      u                        agcagcuguuac    cu     ua          u


6.Novel-miR028
pri_seq                                      uuuagaaauuguguggcuuaaugaaucauaguaauuuuuuguucuuguuauugcuagcgugcuuaaaauauuuccaaugaaugaauaauauuuugaaugauucauugggcucgcacaguuucccaagc
t00873241_x3                                 .....aaauuguguggcuuaaugaau......................................................................................................    0
t03071185_x2                                 ......aauuguguggcuuaauga........................................................................................................    0
t02292180_x1                                 ......aauuguguggcuuaaugaau......................................................................................................    0
t03688332_x238                               ......aauuguguggcuuaaugaauc.....................................................................................................    0
t02225077_x2                                 ......aauuguguggcuuaaugaauca....................................................................................................    0
t01631888_x3                                 ......aauuguguggcuuaaugaaucaua..................................................................................................    0
t04085735_x3                                 ....................................................................................................uucauugggcucgcacag..........    0
t04382356_x6                                 ....................................................................................................uucauugggcucgcacaguu........    0
t03040559_x3                                 ....................................................................................................uucauugggcucgcacaguuu.......    0
t00747994_x538                               ....................................................................................................uucauugggcucgcacaguuuc......    0
t03196339_x2                                 .....................................................................................................ucauugggcucgcacaguuuc......    0
t00416733_x1                                 .....................................................................................................ucauugggcucgcacaguuucc.....    0

--uuua           -              ga   uu u      ugu      cu  c  gc
      gaAAUUGUGUG GCUUAAUGAAUCau  uaa  u uuguuc   uuauug  ag gu  u
      ||||||||||| ||||||||||||||  |||  | ||||||   ||||||  || ||  u
      CUUUGACACGC CGGGUUACUUagua  guu  a aauaag   aguaac  uu ua  a
cgaacc           U              a-   uu u      ua-      cu  a  aa


7.Novel-miR031
pri_seq                                      cuuucgguugggcgucguugugaaaaggcuucucuuugcgccuuagccuuuuccaaugccgcccauaccgauggcuuccgaucuacucuccccuuuc
t02770786_x3                                 .....gguugggcgucguuguga..........................................................................   0
t02703825_x4                                 .....gguugggcgucguugugaa.........................................................................   0
t03891959_x5                                 .....gguugggcgucguugugaaaa.......................................................................   0
t01216550_x113                               .....gguugggcgucguugugaaaag......................................................................   0
t00299301_x2                                 .....gguugggcgucguugugaaaagg.....................................................................   0
t02246478_x3                                 .....gguugggcgucguugugaaaaggc....................................................................   0
t02385795_x9                                 .....gguugggcgucguugugaaaaggcu...................................................................   0
t01170586_x2                                 ........ugggcgucguugugaaaag......................................................................   0
t02960432_x15                                ........ugggcgucguugugaaaaggc....................................................................   0
t02258617_x3                                 ........ugggcgucguugugaaaaggcu...................................................................   0
t03839281_x3                                 ...............................................cuuuuccaaugccgcccauaccga..........................   0
t01770592_x1                                 ................................................uuuuccaaugccgcccauacc............................   0
t01601767_x109                               ................................................uuuuccaaugccgcccauaccga..........................   0
t02985151_x3                                 .................................................uuuccaaugccgcccaua..............................   0
t03817245_x55                                .................................................uuuccaaugccgcccauacc............................   0
t03691907_x29                                .................................................uuuccaaugccgcccauaccg...........................   0
t03626165_x15943                             .................................................uuuccaaugccgcccauaccga..........................   0
t03602315_x12                                .................................................uuuccaaugccgcccauaccgau.........................   0
t02285255_x15                                ..................................................uuccaaugccgcccauaccga..........................   0
t01622970_x1                                 ...................................................uccaaugccgcccauaccga..........................   0

-----------------------  u     -      U     U         ucucuu
                       cu ucGGU UGGGCG CGUUG GAAAAGgcu      u
                       || ||||| |||||| ||||| |||||||||
                       gg AGCCA ACCCGC GUAAC CUUUuccga      g
cuuuccccucucaucuagccuuc  u     U      C     -         uuccgc
```
