# Supplementary figures and images for: Identification and Characterization of Salvia miltiorrhizain miRNAs in Response to Replanting Disease
Source: PLoS One. 2016 Aug 2;11(8):e0159905. doi: 10.1371/journal.pone.0159905 (PMC4970794; doi:10.1371/journal.pone.0159905)

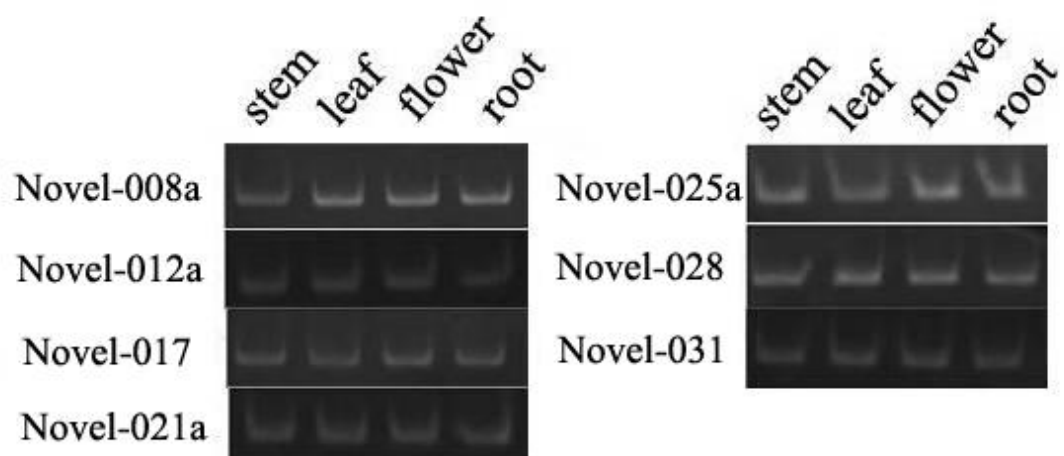

Figure S2: RT-PCR products of the 7 novel miRNAs in the root, stem, leaf and flower of *S. miltiorrhiza*.

Supplement: S2 Fig — (PDF) [file pone.0159905.s002.pdf]
