# Supplementary material for: Identification and Characterization of Salvia miltiorrhizain miRNAs in Response to Replanting Disease
Source: PLoS One. 2016 Aug 2;11(8):e0159905. doi: 10.1371/journal.pone.0159905 (PMC4970794; doi:10.1371/journal.pone.0159905)
Supplement: S3 Fig — A) The miRNAs were significantly upregulated and the targets were no significant differential expression in SPR; B) The miRNAs and the targets were both significantly upregulated in SPR;C) Differential expression miRNAs without matching targets. (PDF) [file pone.0159905.s003.pdf]

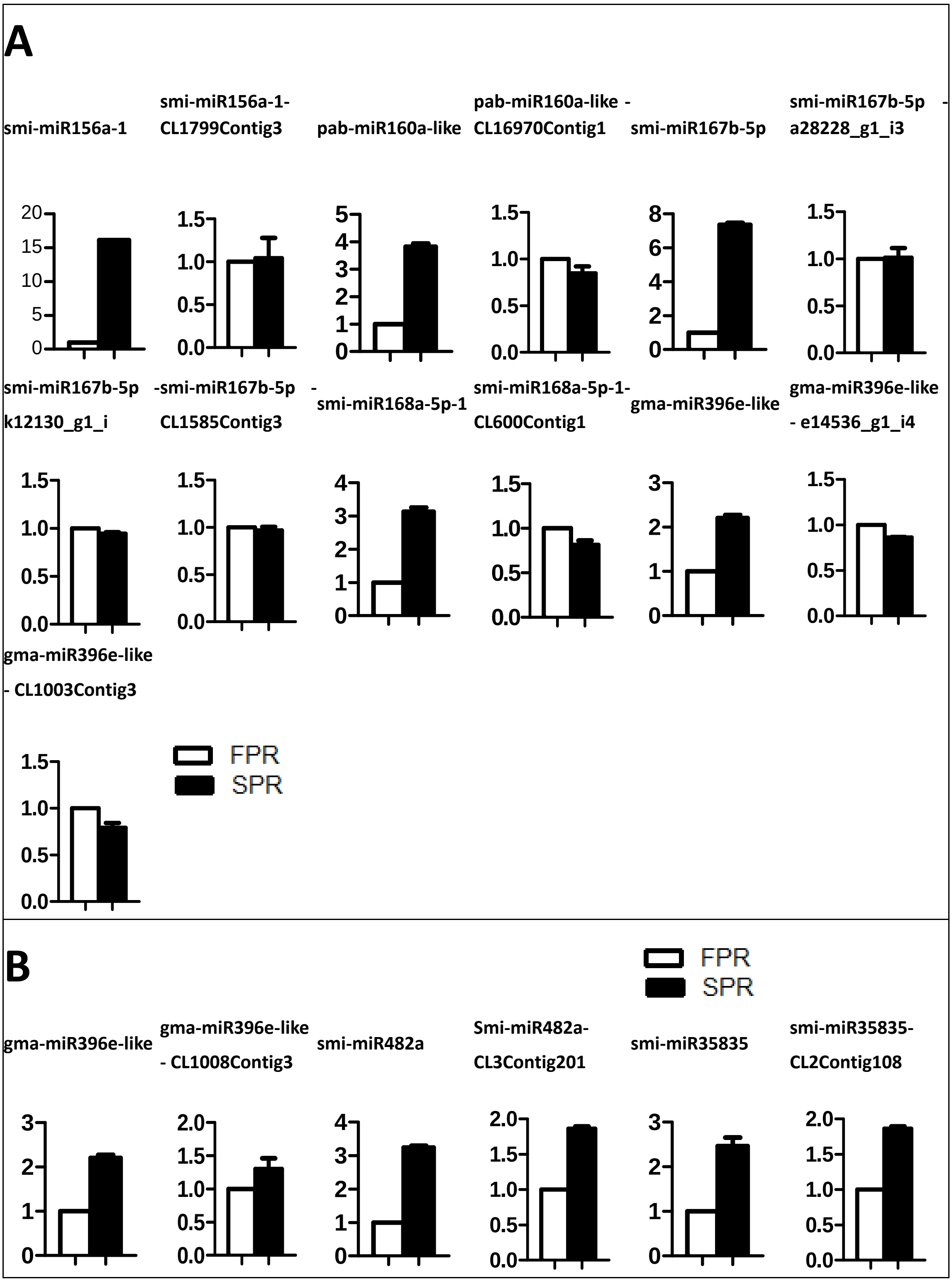
A
smi-miR156a-1
smi-miR156a-1-CL1799Contig3
pab-miR160a-like
pab-miR160a-like - CL16970Contig1
smi-miR167b-5p
smi-miR167b-5p - a28228_g1_i3
smi-miR167b-5p k12130_g1_i
-smi-miR167b-5p - CL1585Contig3
smi-miR168a-5p-1
smi-miR168a-5p-1-CL600Contig1
gma-miR396e-like
gma-miR396e-like - e14536_g1_i4
gma-miR396e-like - CL1003Contig3
FPR
SPR
B
FPR
SPR
gma-miR396e-like
gma-miR396e-like - CL1008Contig3
smi-miR482a
Smi-miR482a-CL3Contig201
smi-miR35835
smi-miR35835-CL2Contig108

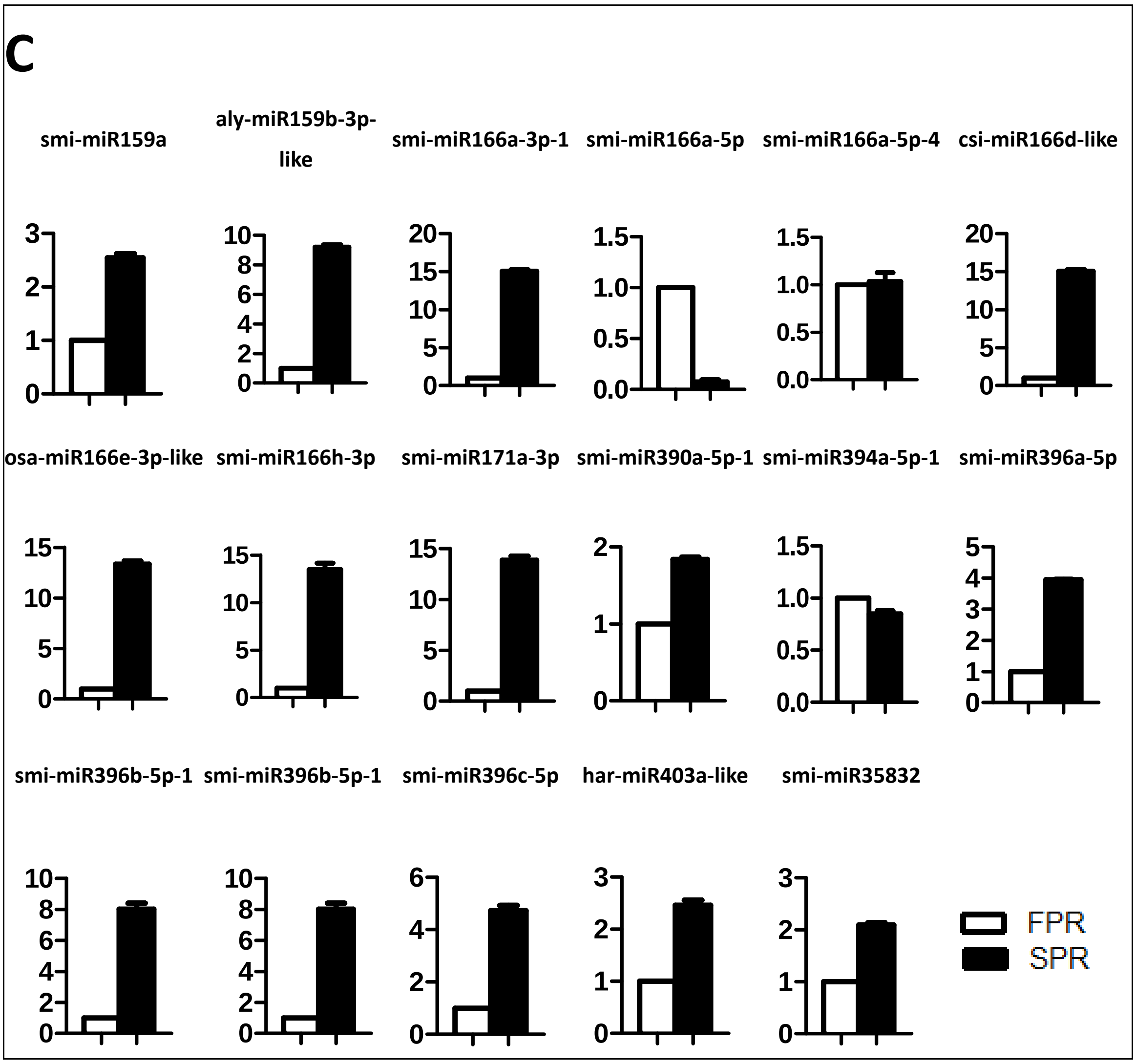

S 3 Fig. Comparison of the expression levels from differential expression miRNAs and the targets between FPR and SPR using qRT-PCR. **A** The miRNAs were significantly upregulated and the targets were no significant differential expression in SPR; **B** The miRNAs and the targets were both significantly upregulated in SPR;**C** Differential expression miRNAs without matching targets.
