# Supplementary material for: Identification and Characterization of Salvia miltiorrhizain miRNAs in Response to Replanting Disease
Source: PLoS One. 2016 Aug 2;11(8):e0159905. doi: 10.1371/journal.pone.0159905 (PMC4970794; doi:10.1371/journal.pone.0159905)
Supplement: S4 Fig — The red dot indicate the cleavage sites. (PPTX) [file pone.0159905.s004.pptx]

## Slide 1
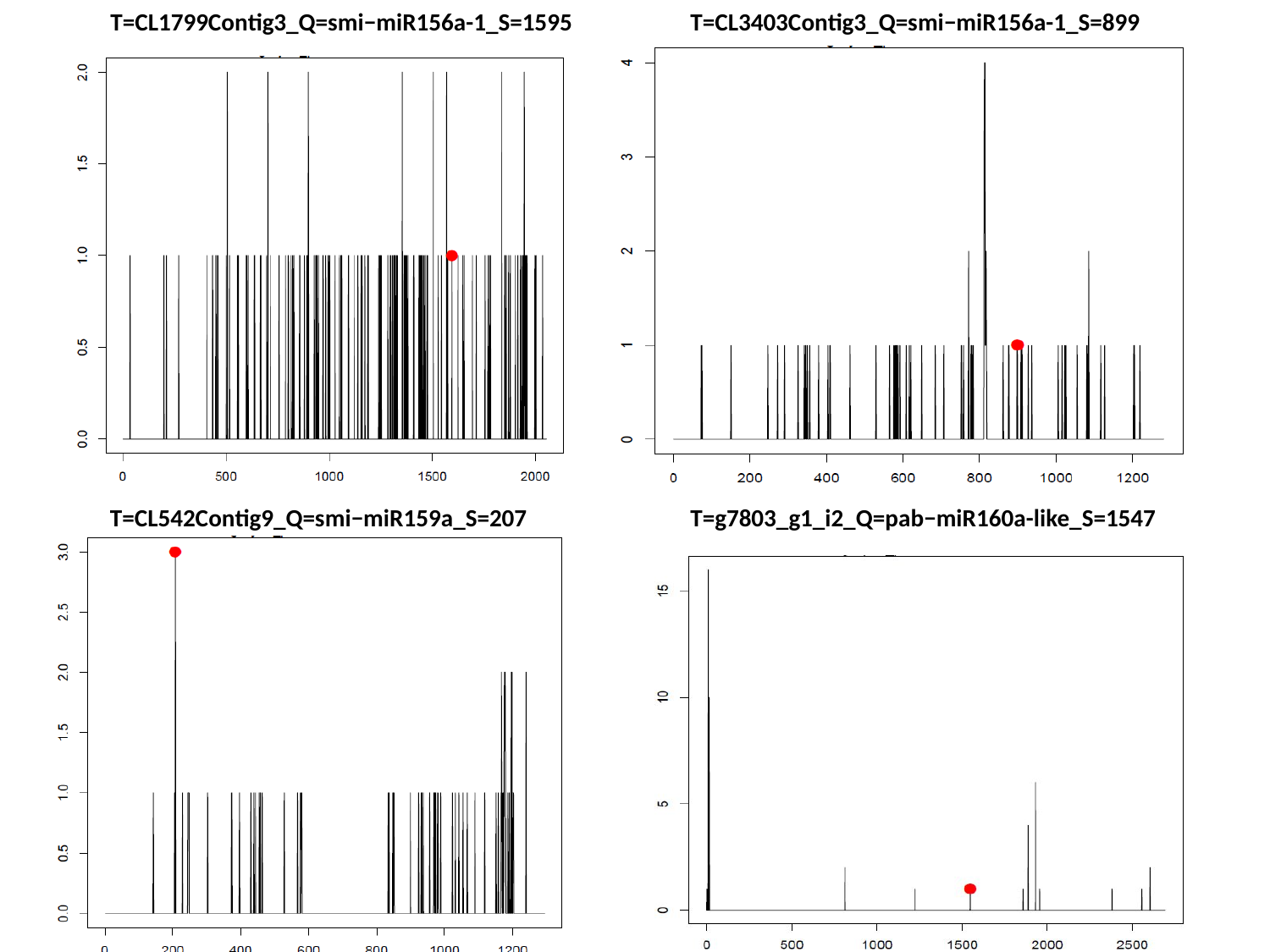

T=CL1799Contig3_Q=smi−miR156a-1_S=1595
T=CL3403Contig3_Q=smi−miR156a-1_S=899
T=CL542Contig9_Q=smi−miR159a_S=207
T=g7803_g1_i2_Q=pab−miR160a-like_S=1547

## Slide 2
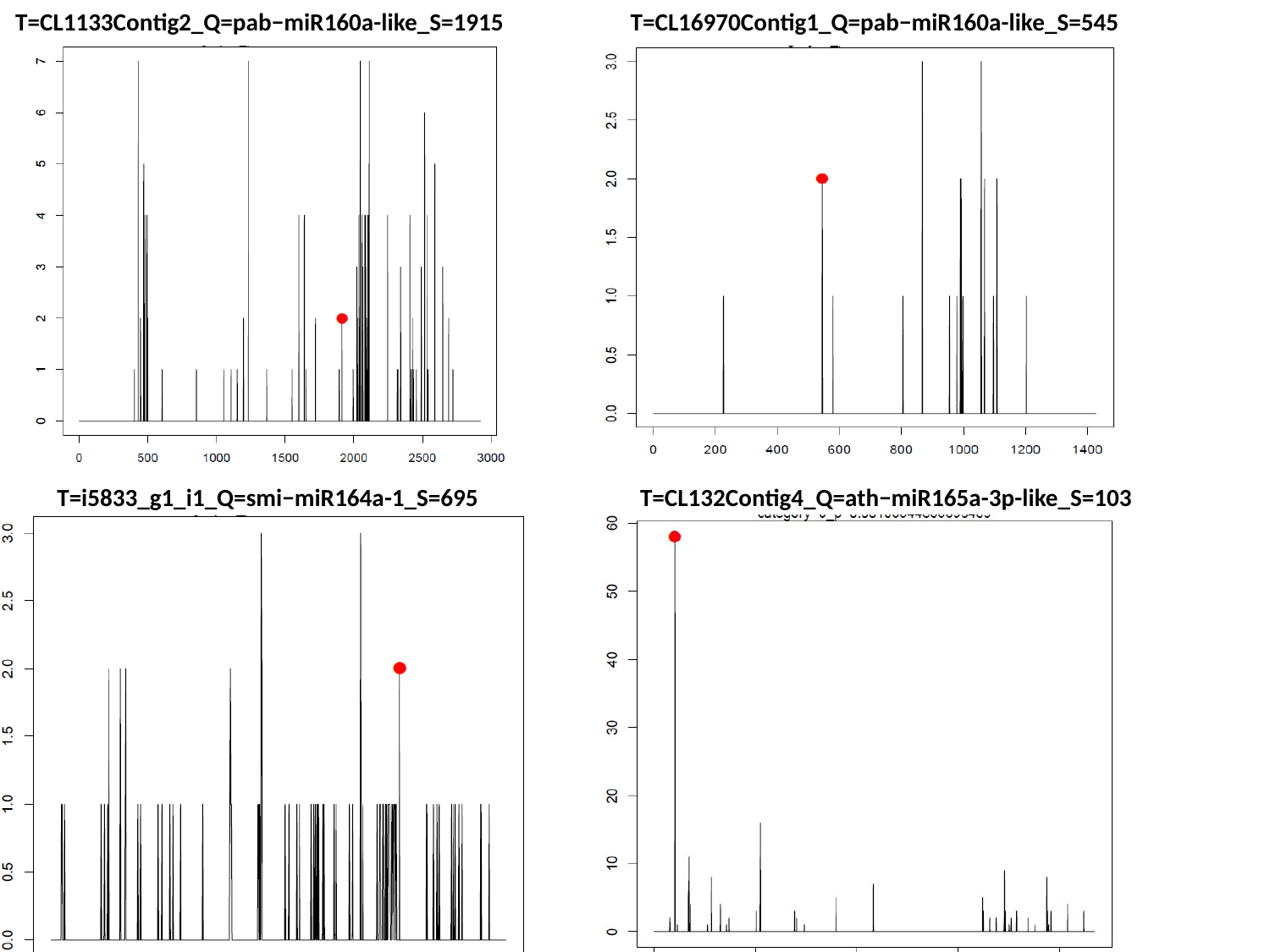

T=CL1133Contig2_Q=pab−miR160a-like_S=1915
T=CL16970Contig1_Q=pab−miR160a-like_S=545
T=i5833_g1_i1_Q=smi−miR164a-1_S=695
T=CL132Contig4_Q=ath−miR165a-3p-like_S=103

## Slide 3
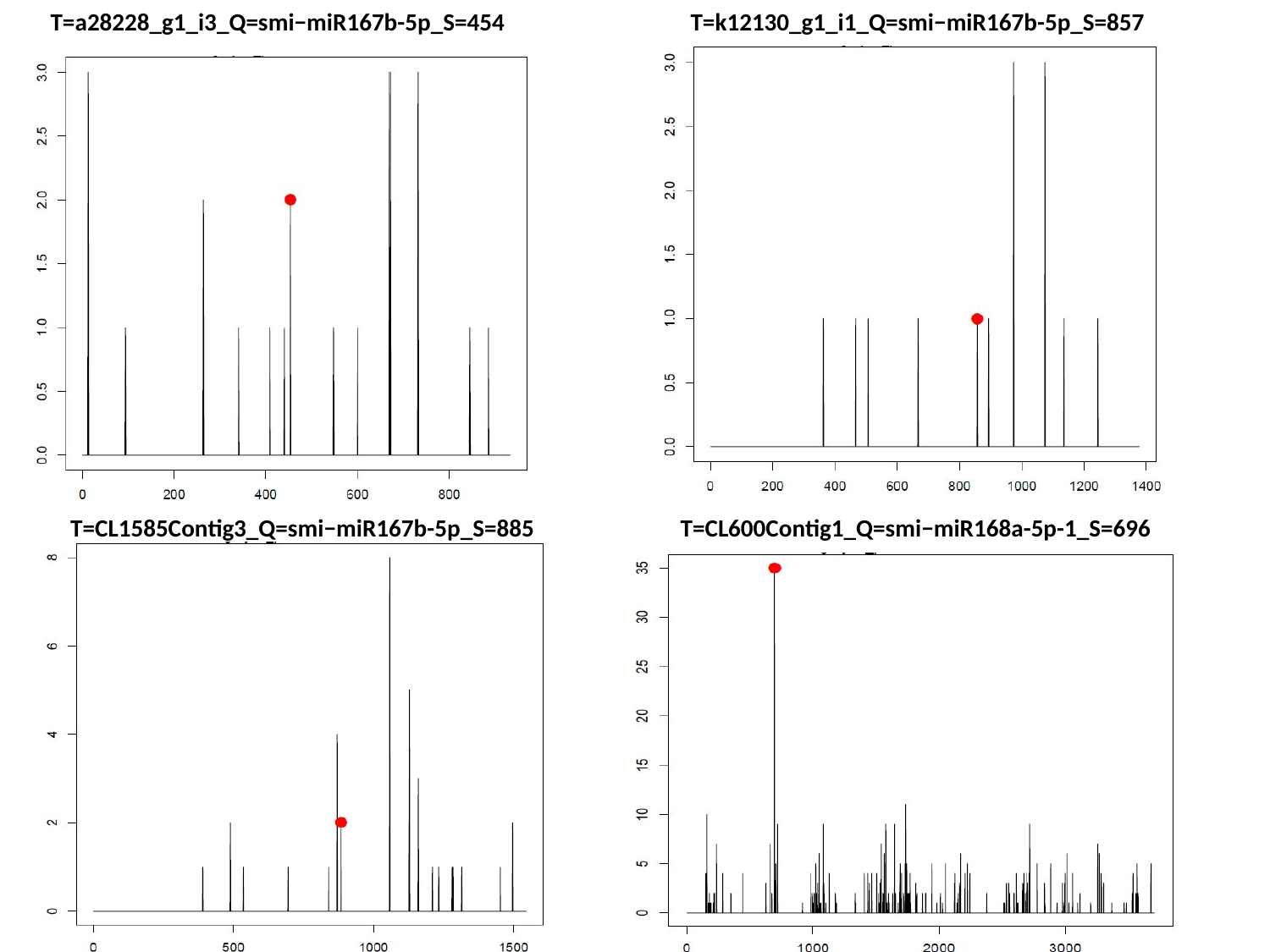

T=a28228_g1_i3_Q=smi−miR167b-5p_S=454
T=k12130_g1_i1_Q=smi−miR167b-5p_S=857
T=CL1585Contig3_Q=smi−miR167b-5p_S=885
T=CL600Contig1_Q=smi−miR168a-5p-1_S=696

## Slide 4
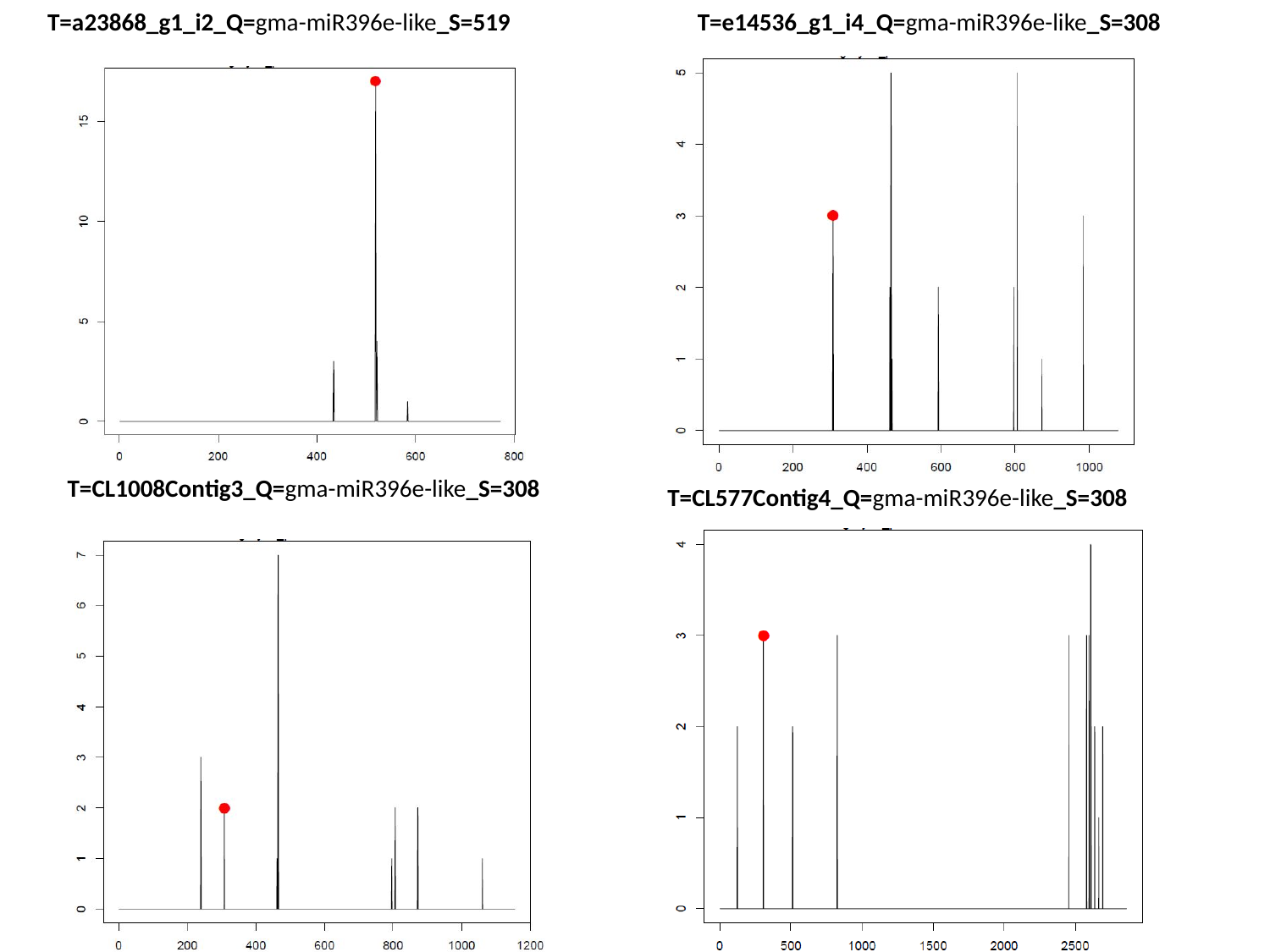

T=a23868_g1_i2_Q=gma-miR396e-like_S=519
T=e14536_g1_i4_Q=gma-miR396e-like_S=308
T=CL1008Contig3_Q=gma-miR396e-like_S=308
T=CL577Contig4_Q=gma-miR396e-like_S=308

## Slide 5
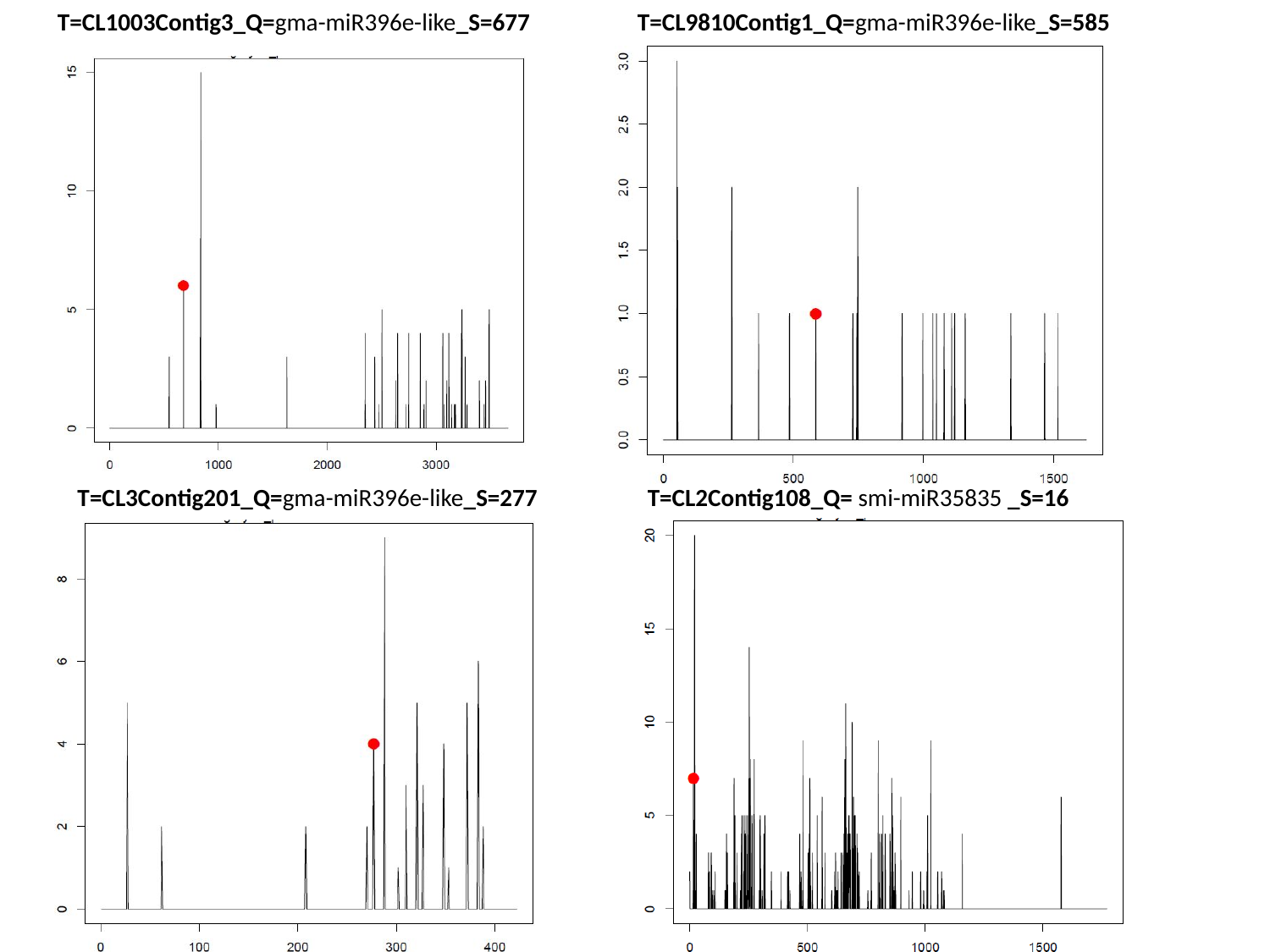

T=CL1003Contig3_Q=gma-miR396e-like_S=677
T=CL9810Contig1_Q=gma-miR396e-like_S=585
T=CL3Contig201_Q=gma-miR396e-like_S=277
T=CL2Contig108_Q= smi-miR35835 _S=16
